# Supplementary material for: Polymorphisms in Pfkelch13 domains before and after the introduction of artemisinin-based combination therapy in Southwest Nigeria
Source: PLoS One. 2025 Mar 31;20(3):e0316479. doi: 10.1371/journal.pone.0316479 (PMC11957316; doi:10.1371/journal.pone.0316479)

Samples: 12634
Bases: 1058
Average spacing: 12



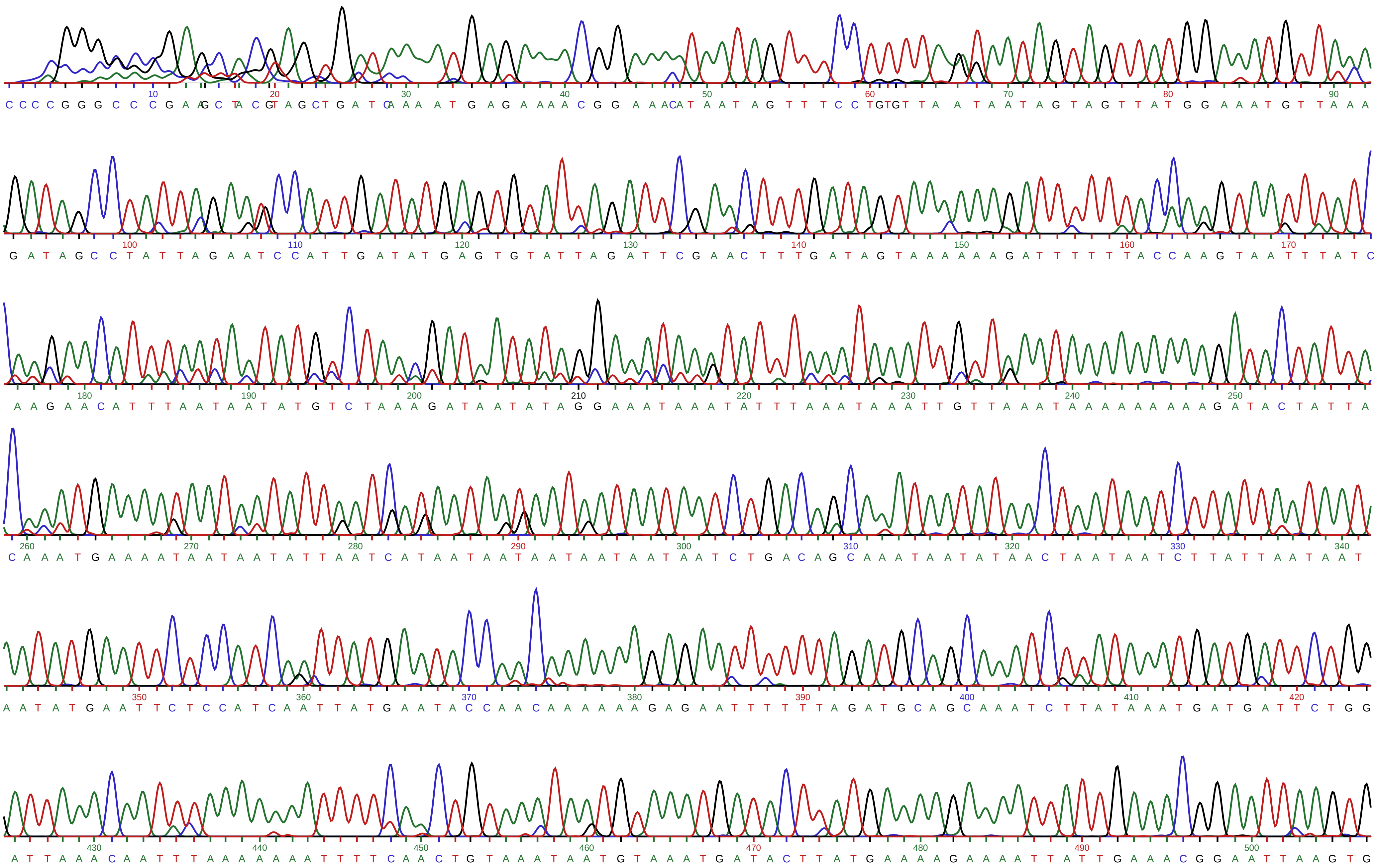

Samples: 12634
Bases: 1058
Average spacing: 12



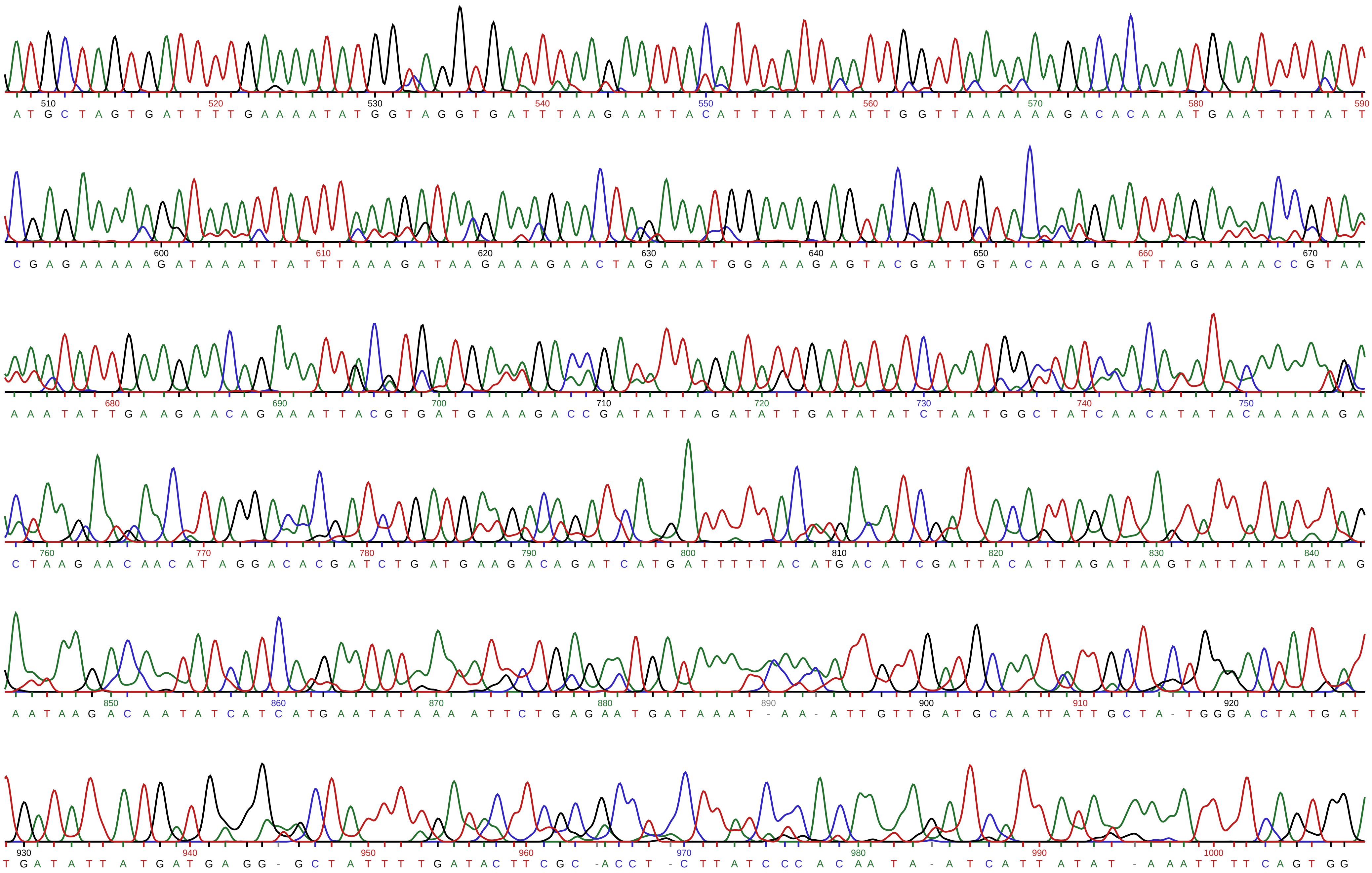

| | |
|---|---|
| Samples: | 12634 |
| Bases: | 1058 |
| Average spacing: | 12 |



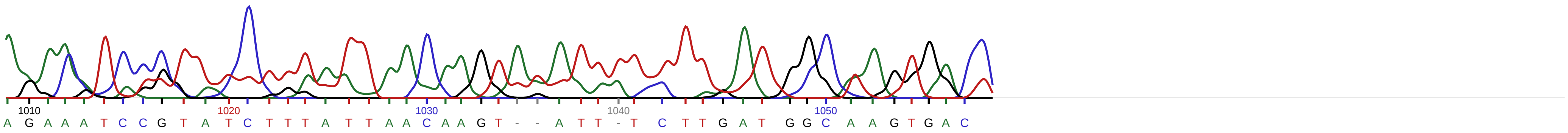

Supplement: Supporting information 2 — (ZIP) [file pone.0316479.s002.zip › 075KN2F_PREMIX_Plate_KELCH2_B10.pdf]
